# Synthesis and Anticancer Activity Evaluation of Novel Phenanthridine Derivatives

**DOI:** 10.3389/fonc.2019.00274

**Published:** 2019-04-16

**Authors:** Minghui Wan, Lei Zhang, Yiming Chen, Qiang Li, Wenli Fan, Qingxia Xue, Fang Yan, Weiguo Song

**Affiliations:** School of Pharmacy, Weifang Medical University, Weifang, China

**Keywords:** phenanthridine, anticancer, topoisomerase, apoptosis, cell cycle arrest

## Abstract

Based on the structure of sanguinarine, fourteen phenanthridine derivatives were designed and synthesized in the current study. The cytotoxic activities of synthesized compounds were evaluated against five human cancer cell lines (MCF-7, PC3, Hela, A549, and HepG2 cell lines) via MTT assay. Among all the compounds tested, molecule **8a** exhibited significant cytotoxic activity against MCF-7 cells with a IC_50_ value of 0.28 μM. A following up enzymatic assay indicated that compound **8a** could inhibit the activity of DNA topoisomerase I/II. Further mechanistic studies performed in the MCF-7 cell line revealed that compound **8a** could arrest cell cycle in S phase and induce cell apoptosis via downregulation of Bcl-2 and upregulation of Bax. Collectively, a potent DNA topoisomerase inhibitor (**8a**) was discovered, which exhibited potential as a candidate chemotherapeutic agent for the management of tumors in the present study.

## Introduction

**Figure 1:**
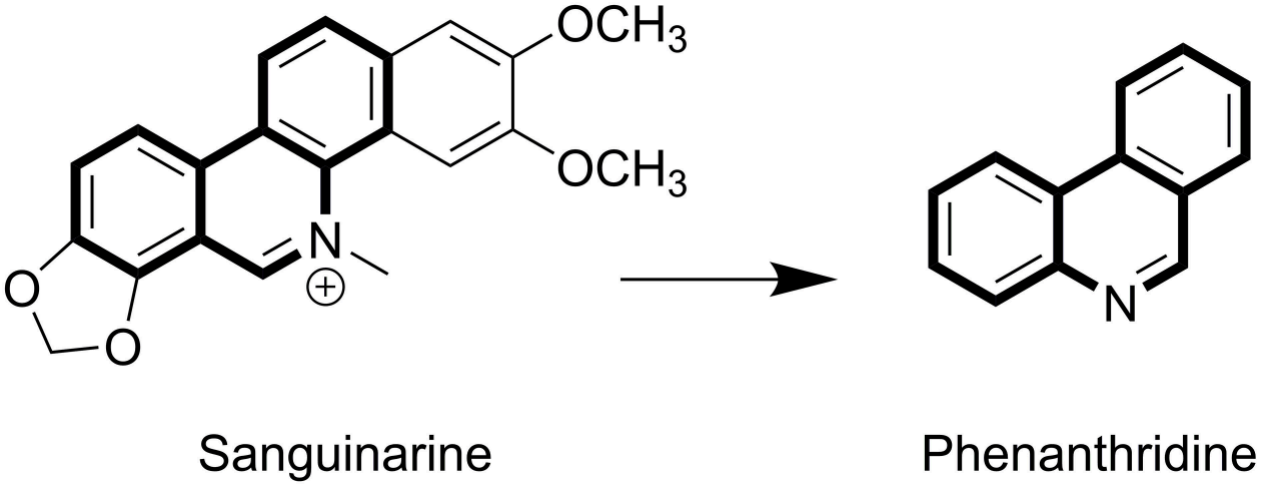
The structures of SA and phenanthridine.

Sanguinarine (SA) belongs to the chrysene-skeleton-based heterocyclic benzo [c] phenanthridine alkaloids family (Figure 1), which are widely distributed in plants, such as *Sanguinaria canadensis* and *Papaveraceae* (1–3). Although SA was isolated in the late 1940s (4), extensive research focusing on the molecular mechanism of its anti-tumor effects has commenced only recently (5). SA has attracted extensive attention because of its significant biological activities, including anti-tumor (6, 7), anti-inflammatory, anti-angiogenesis, antiplatelet, antiviral, and anti-fungal effects (8–11). The flat polyaromatic structure of SA enabled it to directly interact with DNA (12). SA-induced cell cycle arrest and apoptosis was found to not only be caused by DNA damage, but also to be a combined result of targeting other cell structures, such as topoisomerases (Top) (13, 14), antiapoptotic protein (6, 15, 16), and mitochondrial membranes (17, 18).

Previous studies reported that SA might interfere with mitochondrial membranes and induce apoptosis in the CEM leukemia cell line HL-60 (18, 19) and KB carcinoma cell line (17). The potential mechanism was associated with nuclear factor (NF-κB) activation (1), mitochondria damage induced caspase activation (20), and increased expression of Bax/Bcl-2 (21, 22). The proapoptotic effects of SA have significant potential in the development of novel antitumor agents with SA as a lead compound. In addition, SA elicited G0/G1 cell cycle arrest (23), which can be associated with the translocation of cyclin D1 and Top II from nucleus to cytoplasm (24, 25). Additionally, NF-κB, AP-1, MMP-9, and STAT3 inhibition were also observed following SA treatment (26–28) and subsequently resulted in suppressed cancer cell metastasis. Moreover, abolishment of VEGF-induced AKT activation was also proposed as another potential mechanism for the antiangiogenic activity of SA (29, 30), which was believed to contribute to its anti-tumor effects in the animal models of melanoma (31) and colorectal cancer (26).

SA exhibited significant potential in the development of new antitumor drugs, as indicated from the results of a wide range of *in vitro* and *in vivo* investigations. Due to the structure of multiple aromatic rings, further development of SA as antitumor agent is restricted by its low solubilities and severe side effects. To discover SA analogs with improved solubilities and activities, a series of phenanthridine derivatives with reduced aromaticities were designed and synthesized using phenanthridine as a core scaffold. All the derived compounds were identified with ^13^C NMR, ^1^H NMR, HRMS, and biologically evaluated against MCF-7 (human breast cancer), PC3 (human prostatic cancer), Hela (human cervical cancer), A549 (human lung cancer), and HepG2 (human hepatocellular carcinoma) cell lines. During further investigation of the underlying mechanism, molecular techniques such as flow cytometry, hoechst 33258 staining and western blotting were utilized with the representative compounds synthesized in the current study.

### Chemistry

**Scheme 1:**
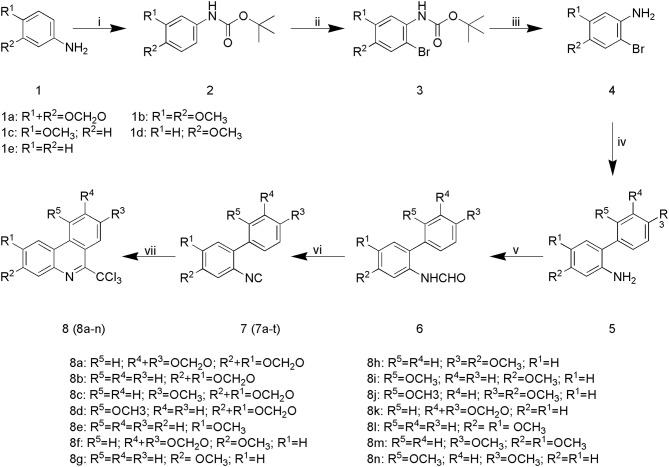
Synthesis of SA derivatives **8a-n**: (i) IPA, Boc_2_O, ice-bath; (ii) ACN, NBS; (iii) DCM, TFA; (iv) [Pd], K_2_CO_3_, DME, 80°C; (v) HCOOH, THF, 60°C; (vi) POCl_3_, NEt_3_, THF, 0°C; (vii) BPO, AcONa, reflux.

The synthetic pathway of phenanthridine derivatives is shown in Scheme 1. As illustrated, amino protection of starting material **1** was performed to afforded compound **2**. The following bromine substitution and deprotection of amino group were carried out to generate intermediate **4**. Preparation of intermediate **5** was performed by Suzuki coupling of 2-bromoaniline derivatives with corresponding phenylboronic acids. Treatment of intermediate **5** under acidic condition yielded compound **6**, and subsequent dehydration of compound **6** afforded 2-isocyanobiphenyls derivatives **7a**-**t**. In the presence of benzoyl peroxide, phenanthridine derivatives **8a**-**n** were derived by reacting of 2-isocyanobiphenyls derivatives with carbon tetrachloride (32).

### Cytotoxicity Assay

The cytotoxicity of synthesized compounds was evaluated against five tumor cell lines (A549, PC3, MCF-7, HepG2, and Hela) via MTT assay. Initially, two doses of each compound (5 and 1 μmol/L) were evaluated. As shown in Table 1, compounds **8a**, **8b**, **8d**, **8e**, **8l**, **8m**, and **8n** exhibited significant inhibitory activities against MCF-7, PC3, and Hela cells at the dose of 5 μmol/L. However, when compared with the lead compound SA, molecule **8d**, **8l**, and **8n** exhibited lower inhibitory activity at the dose of 1 μmol/L.

**Table 1:** The inhibitory activity on tumor cell of phenanthridine derivatives^a^.

**Compound^**b**^**	**MCF-7 (%)**		**PC3 (%)**		**Hela (%)**	
	**5 μM**	**1 μM**	**5 μM**	**1 μM**	**5 μM**	**1 μM**
**8a**	95.66	71.50	91.21	78.47	88.50	58.76
**8b**	93.66	58.29	89.77	64.16	84.90	54.01
**8c**	25.06	19.56	25.32	<5	19.66	16.00
**8d**	83.17	27.48	81.72	43.50	58.92	28.78
**8e**	95.36	60.07	88.65	73.04	83.80	23.52
**8f**	36.73	15.99	15.65	<5	16.26	<5
**8g**	18.23	23.76	<5	<5	<5	<5
**8h**	31.23	16.30	26.40	<5	15.74	8.61
**8i**	16.15	13.60	<5	<5	<5	<5
**8j**	62.18	14.00	64.62	<5	37.46	7.47
**8k**	19.74	32.60	12.81	<5	8.14	<5
**8l**	94.05	34.75	89.37	42.52	81.62	32.25
**8m**	97.83	89.34	95.26	88.75	87.57	80.26
**8n**	72.26	49.63	64.61	<5	30.69	9.72
**SA**	98.29	53.86	96.42	95.60	96.13	64.41
**VP16**	41.84	13.67	38.39	22.39	29.18	17.42

a *Values are average of three determinations and deviation of data results is <20%*.

b *All compounds were dissolved in DMSO for testing*.

Based on the data mentioned above, compounds **8a**, **8b**, **8e**, and **8m** were selected for further test with more doses against the tumor cell lines. The IC_50_ values of these compounds were summarized in Table 2, all the four compounds exhibited potent cytotoxicity against the five tumor cell lines tested compared with the positive control SA and clinically used antitumor drug Etoposide (VP 16). The results indicated that compounds **8a** and **8m** exhibited potent activities against all the tested cancer cell lines. Molecule **8a** (IC_50_ = 0.28 ± 0.08) showed potency of over 6 times higher than SA (IC_50_ = 1.77 ± 0.06) in the inhibition of MCF-7 cells, and molecule **8m** (IC_50_ = 0.39 ± 0.08) exhibited 8.9 times of potency comparing to SA (IC_50_ = 3.49 ± 0.41) in the inhibition of HepG2 cells. Therefore, **8a**, **8b**, **8e**, and **8m** were selected for further mechanistical studies.

**Table 2:** The ${\text{IC}}_{50}^{\text{a}}$ of phenanthridine derivatives.

**Compound**	**IC**_****50****_ **(μM)** ^**a**^				
	**MCF-7**	**PC3**	**Hela**	**A549**	**HepG2**
**8a**	0.28 ± 0.08	0.30 ± 0.06	0.48 ± 0.07	0.89 ± 0.07	0.70 ± 0.09
**8b**	0.77 ± 0.04	0.76 ± 0.01	0.66 ± 0.12	0.85 ± 0.03	1.23 ± 0.08
**8e**	0.61 ± 0.03	0.45 ± 0.04	1.93 ± 0.02	0.89 ± 0.09	2.21 ± 0.14
**8m**	0.24 ± 0.08	0.22 ± 0.04	0.49 ± 0.02	0.85 ± 0.04	0.39 ± 0.08
**SA**	1.77 ± 0.06	1.67 ± 0.33	1.07 ± 0.06	2.68 ± 0.18	3.49 ± 0.41
**VP16**	>10	>10	>10	>10	>10

a *IC_50_ values are represented as mean ±SD (n = 3)*.

### Topoisomerase Inhibition Assay

**Figure 2:**
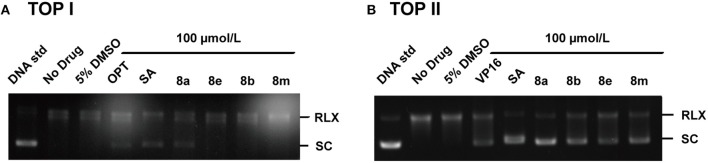
Effects of phenanthridine derivatives and positive control on human Top I **(A)**/IIa **(B)**. Native superhelix pBR322 was incubated at 37°C for 30 min with 2 units of human Top I/IIα in the absence (lane 2) or presence of compound at concentration 100 μM. One hundred micromolar OPT, VP 16, and SA were used as positive controls, respectively. Negatively supercoiled pBR322 (SC) and relaxed DNA (RLX) were shown. DNA samples were run on agarose gel followed by Genecolour I TM staining.

To elucidate the target profiles of the cytotoxic compounds (**8a**, **8b**, **8e**, and **8m**), the inhibitory effects of these compounds were tested against human DNA Top I and IIα by relaxing assay using pBR322 DNA. 10-hydroxy camptothecin (OPT) and VP 16 were used as a positive control for Top I and IIα inhibition, respectively. The Top I/II were able to completely convert the supercoiled DNA to open circular form in the absence of inhibitors (Figure 2, lane B). In contrast, positive control (OPT/VP 16) and active compounds inhibited the activity of Top, which affected the unwinding of the supercoiled DNA, leading to a band pattern similar to the negative control (Figure 2). As shown in Figure 2A, positive control OPT and SA inhibited the activity of both Top I and Top IIα. Compound **8a** exhibited weak Top I inhibition, which was similar to OPT. In the Top IIα test, all the tested compounds exhibited potent DNA Top IIα inhibitory activities at the concentration of 100 μM (Figure 2B). Based on the above findings, molecule **8a** with most potent cytotoxicity and enzymatic inhibitory activities is chosen as a potential candidate for further investigation.

### Cell Cycle Analysis

**Figure 3:**
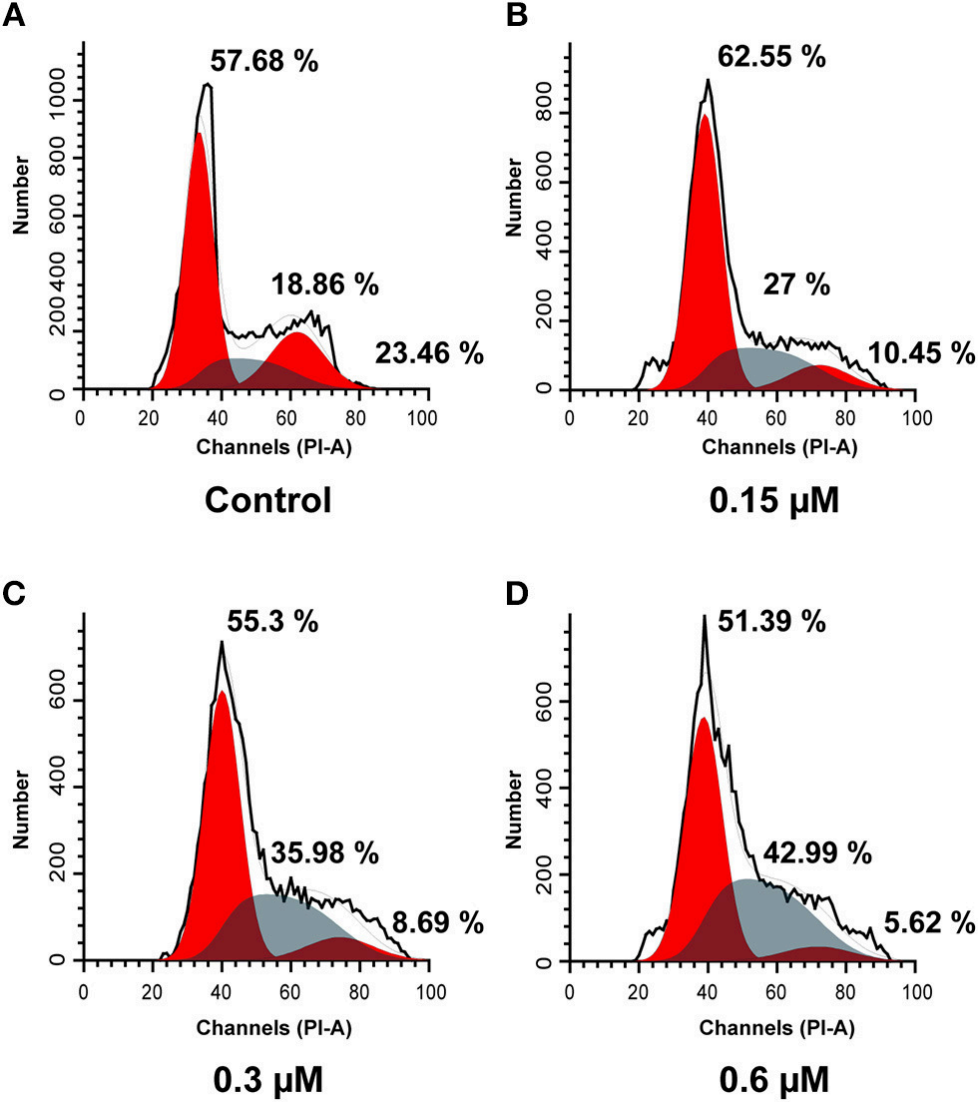
Cell cycle analysis using PI staining of compound **8a** on MCF-7 cells. Cells were treated with compound **8a** at 0.15 **(B)**, 0.3 **(C)**, and 0.6 **(D)** μM for 24 h, compared with the control **(A)**. Cell cycle were detected by flow cytometry.

To elucidate the effects of molecule **8a** on cell cycle distributions, MCF-7 cells were treated with various doses of molecule **8a** (0, 0.15, 0.3, and 0.6 μM) for 24 h. As shown in Figure 3, compound **8a** treatment led to significant accumulation of MCF-7 cells at S phase (from 18.86 to 42.99%) dose-dependently. While reduced cells at the G2/M phase was detected from 23.46 to 10.45% (0.15 μM), 8.69% (0.3 μM), and 5.62% (0.6 μM) following treatment with compound **8a** dose-dependently. These results suggest that compound **8a** exhibited a significant antitumor effect and led to MCF-7 cell cycle arrest at the S phase in a dose-dependent manner.

### Cell Apoptosis Assay

**Figure 4:**
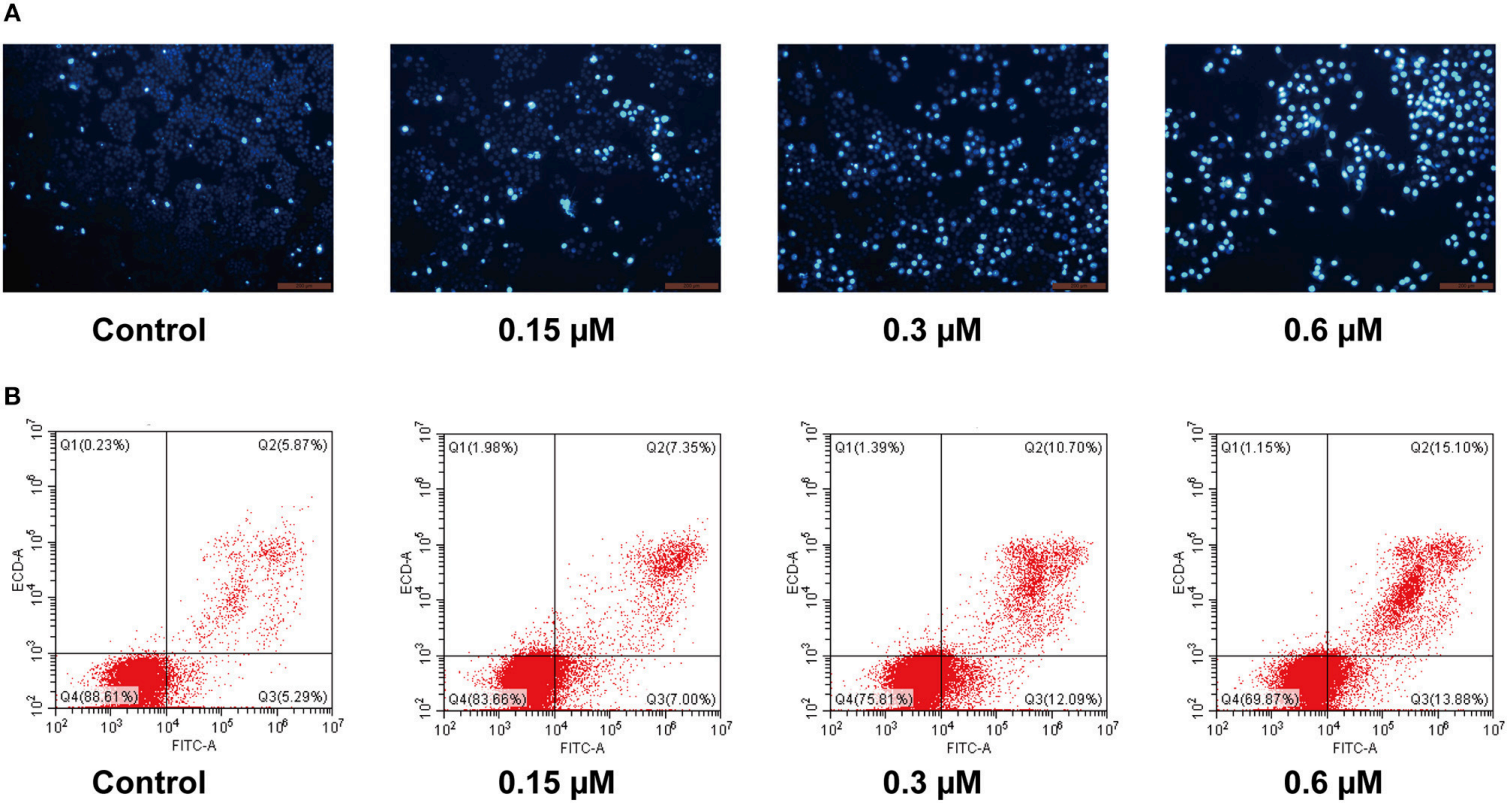
Pro-apoptotic effect of compound **8a** on MCF-7 cells. **(A)** Apoptotic assay by Hoechst 33258. MCF-7 cells were treated with compound **8a** at 0.15, 0.3, and 0.6 μM for 24 h, and then cells were stained with Hoechst 33258 and visualized under a fluorescent microscope. **(B)** Apoptotic assay by flow cytometry. MCF-7 cells were treated with compound **8a** at 0.15, 0.3, and 0.6 μM for 24 h. Then cells were stained with Annexin V-FITC/PI and were detected by flow cytometry analysis.

To further investigate the role of apoptosis in the antitumor effect of compound **8a**, Hoechst 33258 staining was performed to investigate the nuclear morphological changes following molecule **8a** treatment on MCF-7 cells. Hoechst 33258 is a fluorescent stain used to label DNA; live cells nuclei will be stained with uniformly light blue and apoptotic cells nuclei will be stained with bright blue because of chromatin condensation. As shown in Figure 4A, higher levers of apoptotic cells with nuclear condensation, nuclear fragmentation and enhanced brightness were detected in the cells following treatment with various doses of molecule **8a** (0.15, 0.3, and 0.6 μM). To quantify the number of apoptotic cells and to distinguish early apoptosis and secondary necrosis, MCF-7 cells were stained with annexin V-FITC/PI. As shown in Figure 4B, after treatment with difference doses of compound **8a** (0, 0.15, 0.3, and 0.6 μM), the percentage of apoptotic cells were significantly increased from 11.16% of the control to 14.35, 22.79, and 28.98%, respectively, indicating that induction of cell apoptosis contributes to the antitumor effect of compound **8a**.

### Protein Expressions of Bcl-2 and Bax

**Figure 5:**
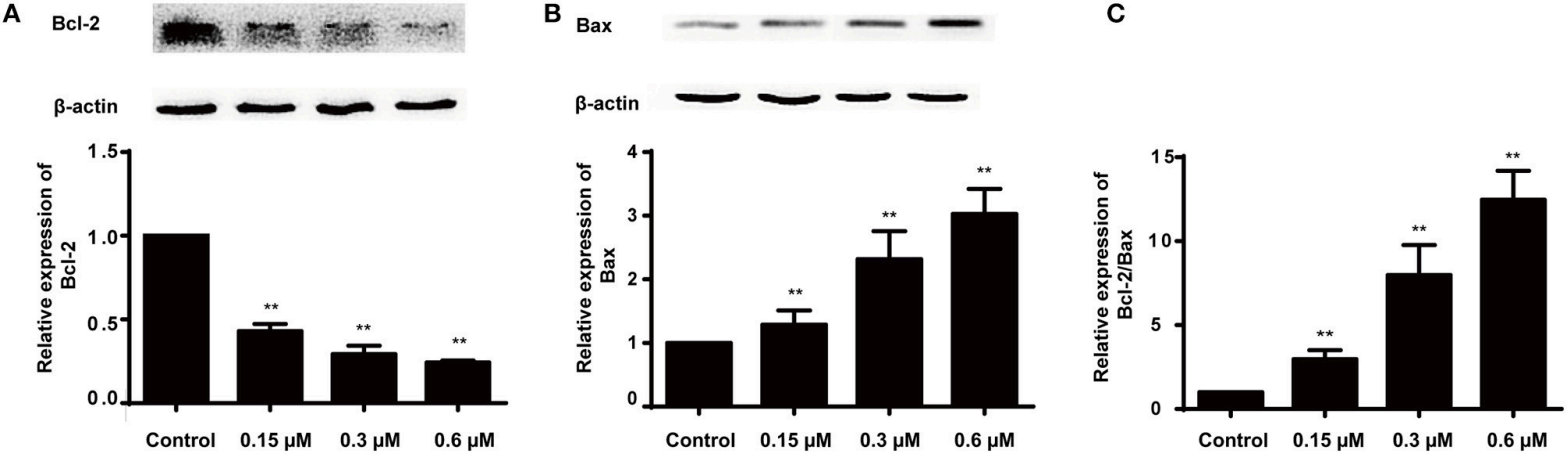
Effects of **8a** on the expressions of Bcl-2 **(A)**, Bax **(B)** and the expression ratio **(C)** in MCF-7 cells. The cells were treated with different concentrations (0.15, 0.3, and 0.6 μM) for 24 h; β-actin served as an internal control. All date were represented as mean ± SD (*n* = 3). **p < 0.01, compared with control group.

Apoptosis is a heavily regulated cell death process influenced by a series of regulatory molecules (33). The mitochondria-dependent pathway has been described as an important signaling pathway of cell apoptosis regulated by the Bcl-2 family including the pro- and anti-apoptotic proteins such as Bax (pro-apoptotic protein) and Bcl-2 (anti-apoptotic protein) (34–36). Moreover, the ratio of Bax/Bcl-2 is important for apoptosis induced by the mitochondrial pathway. Therefore, the effect of compound **8a** on the levels of Bax and Bcl-2 was evaluated in MCF-7 cells. The results indicated that compound **8a** could significantly downregulate Bcl-2 levels and upregulate Bax levels in MCF-7 cells, increasing the ratio of Bax/Bcl-2 in a dose-dependent manner (Figure 5). Collectively, these results suggest that compound **8a** induced apoptosis by regulating the expression of apoptosis-related proteins.

## Conclusions

Based on the structure of sanguinarine, fourteen phenanthridine derivatives **8a**-**m** were synthesized and evaluated for their cytotoxic activity against five different human cancer cell lines. Among the evaluated compounds, **8a** exhibited a broad spectrum of anti-proliferative activities against all the tested cancer cell lines. Further mechanistic assay revealed that compound **8a** could inhibit the activity of both DNA Top I and Top II, as well as preventing cell transition from S to G2 phase dose-dependently. Apoptosis studies against MCF-7 cells indicated that downregulation of Bcl-2 and upregulation of Bax expression may contribute to the anti-proliferative activities. In summary, these findings suggest that molecule **8a** is a potent lead compound in the derived phenanthridine derivatives. Further molecule **8a** based structural modification may be beneficial in the discovery of novel anticancer agents with improved antitumor activity and reduced side effects.

## Materials and Methods

### Chemistry

All chemicals were obtained from commercial suppliers and used without further purification. Reactions progress was detected by thin layer chromatography (TLC) and visualized under UV light. Two hundred to three hundred mesh silica gel was used for column chromatography. All compounds were characterized by ^13^C NMR, ^1^H NMR, and HRMS. ^1^H and ^13^C NMR spectra were recorded on Mercury Plus-400 with internal standard used TMS and recorded in parts per million (ppm). Date were reported as s (singlet), br (broad), s (singlet), d (doublet), t (triplet), q (quartet), m (multiplet), and coupling constant (J) in hertz (Hz). Melting point was determined by MP 100 Automatic Melting Point Apparatus.

#### Representative Procedure for the Synthesis of Compounds 7a-t

To dissolve compound **6**, THF and NEt_3_ was added, the solution was added to POCl_3_ (11 mmol) until the solution was cooled to 0°C. The reaction was quenched by saturated Na_2_CO_3_ until complete consumption of starting material, monitored by TLC. The solution of the crude product was extracted with ethyl acetate, and organic layer was dried over Na_2_SO_4_ and evaporated to dryness. The residue was purified by column chromatography with silica gel (200–300 mesh).

##### 2-isocyano-3′,4′-Methylenedioxy-4,5-methylenedioxy-1,1′-biphenyl (7a)

Yellowish -white solid, Yield 78%; Mp (154.4–156.1°C); ^1^H NMR (400 MHz, CDCl_3_) δ 6.90 (d, *J* = 8.9 Hz, 4H), 6.78 (s, 1H), 6.05 (s, 2H), 6.01 (s, 2H).

##### 2-isocyano-4,5-methylenedioxy-1,1′-biphenyl (7b)

Brown solid, Yield 80%; Mp (90.1–90.3°C); ^1^H NMR (400 MHz, CDCl_3_) δ 7.54–7.30 (m, 5H), 6.91 (s, 1H), 6.82 (s, 1H), 6.05 (s, 2H).

##### 2-isocyano-4,5-methylenedioxy-4′-methoxy-1,1′-biphenyl (7c)

White solid, Yield 81%; Mp (132–133.1°C);^1^H NMR (400 MHz, CDCl_3_) δ 7.39 (d, *J* = 8.7 Hz, 2H), 6.98 (d, *J* = 8.7 Hz, 2H), 6.90 (s, 1H), 6.80 (s, 1H), 6.05 (s, 2H), 3.85 (s, 3H).

##### 2-isocyano-4,5-methylenedioxy-2′-methoxy-1,1′-biphenyl (7d)

Yellowish-white solid, Yield 82%; Mp (139.4–140.7°C); ^1^H NMR (400 MHz, CDCl_3_) δ 7.43–7.34 (m, 1H), 7.20 (dd, *J* = 7.5, 1.8 Hz, 1H), 7.07–6.96 (m, 2H), 6.90 (s, 1H), 6.79 (s, 1H), 6.05 (s, 2H), 3.83 (s, 3H).

##### 2-isocyano-4,5-methylenedioxy-2′,4′-dimethoxy-1,1′-biphenyl (7e)

Brown solid, Yield 79%; Mp (161.4–161.9°C); ^1^H NMR (400 MHz, CDCl_3_) δ 7.10 (s, 1H), 6.88 (s, 1H), 6.77 (s, 1H), 6.56 (dt, *J* = 5.2, 2.5 Hz, 2H), 6.04 (s, 2H), 3.85 (s, 3H), 3.81 (s, 3H).

##### 2-isocyano-3′,4′-methylenedioxy-5-methoxy-1,1′-biphenyl (7f)

Yellowish-white solid, Yield 81%; Mp (119.6–120.1°C); ^1^H NMR (400 MHz, CDCl_3_) δ 7.58 (d, *J* = 8.6 Hz, 1H), 7.12 (d, *J* = 1.7 Hz, 1H), 7.09–6.95 (m, 4H), 6.10 (s, 2H), 3.84 (s, 3H).

##### 2-isocyano-5-methoxy-1,1′-biphenyl (7g)

Black oil, Yield 83%; ^1^H NMR (400 MHz, CDCl_3_) δ 7.62 (d, *J* = 8.7 Hz, 1H), 7.58–7.42 (m, 5H), 7.08–6.99 (m, 2H), 3.85 (s, 3H).

##### 2'-isocyano-3,4-methylenedioxy-1,1′-biphenyl (7h)

Green solid, Yield 85%; Mp (71.6–73.9°C); ^1^H NMR (400 MHz, CDCl_3_) δ 6.02 (s, 2H), 7.02–6.94 (m, 2H), 6.94–6.87 (m, 1H), 7.46 (d, *J* = 9.3 Hz, 1H), 7.43–7.30 (m, 3H).

##### 2'-isocyano-2,4-dimethoxy-1,1′-biphenyl (7i)

Yellowish-white solid, Yield 79%; Mp (90.1–90.5°C); ^1^H NMR (400 MHz, CDCl_3_) δ 7.41 (ddd, *J* = 8.9, 7.4, 1.8 Hz, 2H), 7.38–7.29 (m, 2H), 7.18–7.10 (m, 1H), 6.58 (dd, *J* = 5.7, 2.2 Hz, 2H), 3.86 (s, 3H), 3.81 (s, 3H).

##### 2′-isocyano-2,4,5′-trimethoxy-1,1′-biphenyl (7j)

Yellow solid, Yield 80%; Mp (104.6–104.9°C); ^1^H NMR (400 MHz, CDCl_3_) δ 7.35 (d, *J* = 8.4 Hz, 1H), 7.14 (d, *J* = 8.9 Hz, 1H), 6.88–6.79 (m, 2H), 6.58 (dd, *J* = 5.4, 2.3 Hz, 2H), 3.86 (s, 3H), 3.82 (s, 6H).

##### 2-isocyano-3′,4′-methylenedioxy-4-methoxy-1,1′-biphenyl (7k)

Yellowish-white solid, Yield 75%; Mp (120.6–120.9°C); ^1^H NMR (400 MHz, CDCl_3_) δ 7.28 (d, *J* = 8.4 Hz, 1H), 7.02–6.86 (m, 5H), 6.02 (s, 2H), 3.85 (s, 3H).

##### 2-isocyano-4-methoxy-1,1′-biphenyl (7l)

Yellow solid, Yield 78%; Mp (117.3–117.6°C); ^1^H NMR (400 MHz, CDCl_3_) δ 7.53–7.43 (m, 4H), 7.43–7.36 (m, 1H), 7.33 (d, *J* = 9.0 Hz, 1H), 7.05–6.97 (m, 2H), 3.86 (s, 3H).

##### 2-isocyano-4,4′-dimethoxy-1,1′-biphenyl (7m)

Yellowish brown solid, Yield 83%; Mp (102.4–102.8°C); ^1^H NMR (400 MHz, CDCl_3_) δ 7.44–7.37 (m, 2H), 7.30 (d, *J* = 9.0 Hz, 1H), 6.99 (d, *J* = 9.0 Hz, 4H), 3.85 (d, *J* = 4.0 Hz, 6H).

##### 2-isocyano-2′,4-dimethoxy-1,1′-biphenyl (7n)

White solid, Yield 82%; Mp (125.5–126°C); ^1^H NMR (400 MHz, CDCl_3_) δ 7.48–7.34 (m, 1H), 7.33–7.17 (m, 2H), 7.08–6.94 (m, 4H), 3.84 (d, *J* = 5.4 Hz, 6H).

##### 2-isocyano-2′,4,4′-trimethoxy-1,1′-biphenyl (7o)

Yellowish-white solid, Yield 80%; Mp (105.9–107.3°C); ^1^H NMR (400 MHz, CDCl_3_) δ 7.26 (d, *J* = 1.7 Hz, 1H), 7.12 (d, *J* = 8.9 Hz, 1H), 7.01–6.93 (m, 2H), 6.61–6.53 (m, 2H), 3.90–3.79 (m, 9H).

##### 2-isocyano-3′,4′-methylenedioxy-4,5-dimethoxy-1,1′-biphenyl (7p)

Brown solid, Yield 84%; Mp (171.7–172.3°C); ^1^H NMR (400 MHz, CDCl_3_) δ 6.99–6.86 (m, 4H), 6.80 (s, 1H), 6.02 (s, 2H), 3.91 (d, *J* = 2.5 Hz, 6H).

##### 2-isocyano-4,5-dimethoxy-1,1′-biphenyl (7q)

Yellowish-white solid, Yield 82%; Mp (139.4–139.9°C); ^1^H NMR (400 MHz, CDCl_3_) δ 7.27 (s, 1H), 7.01 (s, 1H), 3.85 (d, *J* = 3.7 Hz, 5H), 7.58–7.47 (m, 3H), 7.47–7.39 (m, 1H).

##### 2-isocyano-4,4′,5-trimethoxy-1,1′-biphenyl (7r)

Yellowish brown solid, Yield 84%; Mp (102.7–103.7°C); ^1^H NMR (400 MHz, CDCl_3_) δ 7.43 (d, *J* = 8.7 Hz, 2H), 7.00 (d, *J* = 8.7 Hz, 2H), 6.93 (s, 1H), 6.82 (s, 1H), 3.92 (d, *J* = 1.5 Hz, 6H), 3.86 (s, 3H).

##### 2-isocyano-2′,4,5-trimethoxy-1,1′-biphenyl (7s)

Yellow solid, Yield 83%; Mp (103–103.6°C); ^1^H NMR (400 MHz, CDCl_3_) δ 7.44–7.35 (m, 1H), 7.28–7.20 (m, 1H), 7.09–6.98 (m, 2H), 6.94 (s, 1H), 6.82 (s, 1H), 3.94–3.82 (m, 9H).

##### 2-isocyano-2′,4,4′,5-tetramethoxy-1,1′-biphenyl (7t)

Yellowish-white solid, Yield 82%; Mp (123.4–123.9°C); ^1^H NMR (400 MHz, CDCl_3_) δ 7.15 (d, *J* = 8.9 Hz, 1H), 6.92 (s, 1H), 6.79 (s, 1H), 6.57 (dq, *J* = 4.2, 2.4 Hz, 2H), 3.91 (s, 3H), 3.88 (s, 3H), 3.86 (s, 3H), 3.82 (s, 3H).

#### Representative Procedure for the Synthesis of Compounds 8a-8n

A mixture was produced of 2-isocyanobiphenyls derivatives (0.5 mmol), benzoyl peroxide (0.6 mmol), AcONa (1.0 mmol) in CCl_4_ (2 mL) under an atmosphere of N_2_. The reaction was stirred under reflux until complete consumption of starting material, monitored by TLC (about 16h). The solution of the crude product was extracted with ethyl acetate. The organic layers were washed with a saturated solution of NaHCO_3_ and dried over Na_2_SO_4_ and evaporated to dryness. The residue was purified by column chromatography with silica gel (200–300 mesh) to afford the product 6-trichloromethylphenanthridine.

##### 2,3-methylenedioxy-8,9-methylenedioxy-6-(trichloromethyl)phenanthridine (8a)

Yellow solid, Yield 40%; Mp (198.7–199.6°C); ^1^H NMR (400 MHz, CDCl_3_): δ 8.23 (s, 1H), 7.77 (s, 1H), 7.67 (s, 1H), 7.53 (s, 1H), 6.18 (d, *J* = 8.5 Hz, 4H); ^13^C NMR(101 MHz, DMSO): δ 151.43, 150.27, 149.97, 149.09, 148.77, 147.57, 133.32, 129.68, 128.98, 115.89, 107.21, 104.01, 103.18, 102.90, 101.57, 100.33; HRMS (ESI) *m/z* 383.9592 (M+H).

##### 2,3-methylenedioxy-6-(trichloromethyl)phenanthridine (8b)

Yellowish solid, Yield 39%; Mp (175.4–176.5°C); ^1^H NMR(400 MHz, CDCl_3_): δ 8.92 (d, *J* = 8.4 Hz, 1H), 8.49 (d, *J* = 8.2 Hz, 1H), 7.91–7.78 (m, 2H), 7.68 (t, *J* = 7.6 Hz, 1H), 7.60 (s, 1H), 6.19 (s, 2H); ^13^C NMR(101 MHz, DMSO): δ 150.59, 150.37, 150.29, 137.46, 134.81, 131.31, 127.60, 127.04, 124.18, 121.88, 119.36, 107.77, 103.08, 100.50; HRMS (ESI) *m/z* 339.9696 (M+H).

##### 2,3-methylenedioxy-8-methoxy-6-(trichloromethyl)phenanthridine (8c)

Brown solid, Yield 41%; Mp (93.8–95.0°C); ^1^H NMR(400 MHz, CDCl_3_) δ 8.12–8.05 (m, 8H), 7.80 (s, 1H), 7.67 (t, *J* = 7.5 Hz, 4H), 6.17 (s, 2H), 4.01 (s, 3H); ^13^C NMR(101 MHz, DMSO) δ 167.67, 163.08, 162.77, 135.60, 134.08, 133.34, 131.07, 130.82, 129.93, 129.87, 129.78, 129.70, 129.31, 129.01, 128.45, 124.97; HRMS (ESI) *m/z* 369.9804 (M+H).

##### 2,3-methylenedioxy-10-methoxy-6-(trichloromethyl)phenanthridine(8d)

Yellow solid; Yield 38%; Mp (219.7–222.3°C); ^1^H NMR (400 MHz, DMSO) δ 7.95 (d, *J* = 6.3 Hz, 1H), 7.81 (t, *J* = 8.3 Hz, 1H), 7.59 (d, *J* = 3.6 Hz, 2H), 7.31 (t, *J* = 7.8 Hz, 1H), 6.32 (s, 2H), 3.69 (s, 3H); HRMS (ESI) *m/z* 369.9804 (M+H).

##### 2-methoxy-6-(trichloromethyl)phenanthridine (8e)

Yellowish-white solid; Yield 37%; Mp (119–120.9°C); ^1^H NMR (400 MHz, CDCl_3_): δ 8.96 (d, *J* = 8.2 Hz, 1H), 8.65 (d, *J* = 8.4 Hz, 1H), 8.19 (d, *J* = 9.0 Hz, 1H), 7.94–7.83 (m, 2H), 7.80–7.71 (m, 1H), 7.42 (dd, *J* = 9.0, 2.7 Hz, 1H), 4.05 (s, 3H); ^13^C NMR (101 MHz, DMSO): δ 160.62, 149.93, 135.31, 134.27, 132.51, 131.38, 128.05, 127.82, 126.70, 124.59, 120.53, 120.27, 104.00, 98.71, 56.46; HRMS (ESI) *m/z* 325.9901 (M+H).

##### 8,9-methylenedioxy-3-methoxy-6-(trichloromethyl)phenanthridine (8f)

Brown solid; Yield 32%; Mp (197.5–197.8°C); ^1^H NMR (400 MHz, CDCl_3_) δ 8.36–8.19 (m, 2H), 7.91 (s, 1H), 7.59 (d, *J* = 2.7 Hz, 1H), 7.35 (dd, *J* = 9.1, 2.7 Hz, 1H), 6.20 (s, 2H), 4.00 (s, 3H); ^13^C NMR (101 MHz, DMSO): δ 160.32, 151.87, 141.76, 133.80, 124.77, 120.86, 119.65, 115.39, 109.96, 108.77, 104.45, 103.88, 103.25, 101.29, 100.64, 56.08; HRMS (ESI) *m/z* 369.9798 (M+H).

##### 3-methoxy-6-(trichloromethyl)phenanthridine (8g)

Yellow solid; Yield 26%; Mp (175.1–175.3°C); ^1^H NMR (400 MHz, CDCl_3_): δ 8.93 (d, *J* = 8.7 Hz, 1H), 8.62 (d, *J* = 8.4 Hz, 1H), 8.48 (d, *J* = 9.1 Hz, 1H), 7.90–7.81 (m, 1H), 7.72–7.63 (m, 2H), 7.44–7.36 (m, 1H), 4.02 (s, 3H). ^13^C NMR (101 MHz, DMSO): δ 160.81, 152.92, 142.01, 135.11, 131.94, 127.91, 126.82, 124.58, 123.65, 120.95, 119.09,119.06, 110.67, 98.60, 56.13. HRMS (ESI) *m/z* 325.9899 (M+H).

##### 3,8-dimethoxy-6-(trichloromethyl)phenanthridine (8h)

Yellow solid; Yield 40%; Mp (146.7–147.2°C); ^1^H NMR (400 MHz, CDCl_3_): δ 8.52 (d, *J* = 9.2 Hz, 1H), 8.39 (d, *J* = 9.1 Hz, 1H), 8.28 (d, *J* = 2.6 Hz, 1H), 7.63 (d, *J* = 2.7 Hz, 1H), 7.50 (dd, *J* = 9.2, 2.6 Hz, 1H), 7.38 (dd, *J* = 9.1, 2.7 Hz, 1H), 4.01 (d, *J* = 2.4 Hz, 6H); ^13^C NMR (101 MHz, DMSO): δ 160.06, 157.07, 151.84, 141.12, 129.60, 125.46, 124.08, 122.20, 121.18, 120.32, 119.35, 110.40, 108.81, 56.07, 55.91; HRMS (ESI) *m/z* 356.0009 (M+H).

##### 3,10-dimethoxy-6-(trichloromethyl)phenanthridine (8i)

Yellow solid; Yield 37%; Mp (149.9–150.9°C). ^1^H NMR (400 MHz, CDCl_3_): δ 9.46 (d, *J* = 9.5 Hz, 1H), 8.60 (d, *J* = 8.5 Hz, 1H), 7.72–7.56 (m, 2H), 7.41–7.29 (m, 2H), 4.16 (s, 3H), 4.02 (s, 3H); ^13^C NMR (101 MHz, DMSO) δ 159.78, 157.90, 152.59, 142.64, 129.33, 127.12, 125.28, 120.88, 120.27, 120.03, 118.54, 113.19, 110.92, 56.63, 55.97; HRMS (ESI) *m/z* 356.0009 (M+H).

##### 3,8,10-trimethoxy-6-(trichloromethyl)phenanthridine (8j)

Yellow solid; Yield 35%; Mp (97–97.3°C); ^1^H NMR (400 MHz, CDCl_3_): δ 7.98 (d, *J* = 2.3 Hz, 1H), 7.70–7.60 (m, 3H), 7.35 (dd, *J* = 9.5, 2.9 Hz, 1H), 4.12 (s, 3H), 4.01 (d, *J* = 2.9 Hz, 6H); ^13^C NMR (101 MHz, DMSO): δ 162.77, 159.25, 159.09, 157.44, 135.58, 130.82, 129.78, 129.00, 128.38, 120.61, 120.42, 110.71, 103.67, 101.47, 56.84, 55.95, 55.92; HRMS (ESI) *m/z* 386.0112 (M+H).

##### 8,9-methylenedioxy-6-(trichloromethyl)phenanthridine (8k)

Yellowish solid; Yield 32%; Mp (164.4–165°C); ^1^H NMR (400 MHz, CDCl_3_) δ 8.43–8.36 (m, 1H), 8.30 (s, 1H), 8.27–8.20 (m, 1H), 8.02 (s, 1H), 7.73 (tt, *J* = 7.1, 5.3 Hz, 2H), 6.24 (d, *J* = 16.4 Hz, 2H); ^13^C NMR (101 MHz, DMSO) δ 151.82, 151.22, 148.15, 140.03, 133.36, 130.68, 129.57, 125.30, 123.39, 118.56, 116.49, 114.73, 104.69, 103.41, 101.84; HRMS (ESI) *m/z* 339.9697 (M+H).

##### 2,3-dimethoxy-6-(trichloromethyl)phenanthridine (8l)

Yellow solid; Yield 43%; Mp (174.5–176.1°C); ^1^H NMR (400 MHz, CDCl_3_): δ 8.95 (d, *J* = 8.6 Hz, 1H), 8.58 (d, *J* = 8.4 Hz, 1H), 8.16–8.08 (m, 4H), 7.86 (s, 2H), 7.74–7.58 (m, 4H), 7.49 (t, *J* = 7.8 Hz, 4H), 4.16 (s, 3H), 4.11 (s, 3H); ^13^C NMR (101 MHz, DMSO): δ 167.75, 151.93, 151.86, 133.29, 131.16, 129.69, 128.99, 127.70, 126.77, 124.17, 119.98, 119.33, 110.56, 102.99, 56.68, 56.30; HRMS (ESI) *m/z* 356.0010 (M+H).

##### 2,3,8-trimethoxy-6-(trichloromethyl)phenanthridine (8m)

Yellow solid; Yield 39%; Mp (125.5–126.9°C); ^1^H NMR (400 MHz, CDCl_3_): δ 8.48 (d, *J* = 9.1 Hz, 1H), 8.29 (d, *J* = 2.5 Hz, 1H), 7.77 (s, 1H), 7.62 (s, 1H), 7.50 (dd, *J* = 9.2, 2.6 Hz, 1H), 4.14 (s, 3H), 4.09 (s, 3H), 4.02 (s, 3H); ^13^C NMR (101 MHz, DMSO): δ 157.06, 152.00, 151.26, 149.05, 135.36, 129.09, 128.95, 126.01, 121.63, 120.63, 120.29, 110.36, 108.35, 102.47, 56.64, 56.23, 55.89; HRMS (ESI) *m/z* 386.0115 (M+H).

##### 8,10-dimethoxy-6-(trichloromethyl) phenanthridine (8n)

Yellow solid; Yield 39%; Mp (161.3–162°C); ^1^H NMR (400 MHz, CDCl_3_): δ 9.45–9.38 (m, 1H), 8.28–8.21 (m, 1H), 8.01 (d, *J* = 2.3 Hz, 1H), 7.71 (dd, *J* = 6.5, 3.5 Hz, 2H), 7.00 (d, *J* = 2.4 Hz, 1H), 4.13 (s, 3H), 4.02 (s, 3H); ^13^C NMR (101 MHz, DMSO): δ 160.02, 158.37, 151.25, 139.93, 130.95, 130.05, 128.51, 127.15, 124.74, 122.76, 119.71, 103.62, 101.98, 99.01, 56.93, 56.04; HRMS (ESI) *m/z* 356.0007 (M+H).

### Pharmacology

#### Cell Culture

A549, PC3, MCF-7, HepG2 and Hela cell lines were obtained from the Chinese Academy of Sciences Cell Bank. A549, Hela and PC3 were cultured in RPMI-1640 medium supplemented with 10% FBS, MCF-7 cells were maintained in MEM medium supplemented with 10% FBS, HepG2 cells were cultured in DMEM medium supplemented with 10% FBS. All the cell lines were cultured at humidified atmosphere containing 5% CO_2_ at 37°C. The stock solutions (20 mM) of phenanthridine derivatives were prepared in DMSO and added at desired concentrations to the cell culture. DMSO concentration did not exceed 1:1,000 in the final culture.

#### MTT Assay

Cytotoxic activities of the phenanthridine derivatives was evaluated by MTT assay. The stock solutions of phenanthridine derivatives were diluted with culture medium. The cells were seeded in 96-well plates at a density 5 × 10^3^ cells per well and incubated until confluency 90–95%, then each well was treated with 100 μL medium containing the desired concentrations of phenanthridine derivatives and incubated for 48 h. 20 μL MTT working solution (5 mg/mL) was then added to each well and incubated for another 4 h. At the end of incubation, the medium was carefully removed, and 200 μL DMSO was added. The optical density at 490 nm and 630 nm were then measured with a microplate reader (MODEL). The percentage of cell growth inhibition was calculated with the following equation: % inhibition = [1–(Sample group OD_490_ - Sample group OD_630_)/(Control group OD_490_-Control group OD_630_)] × 100%. The IC_50_ values were calculated with Origin 7.5 software, and standard deviations of the IC_50_ values were obtained from at least 3 independent experiments.

#### DNA Top I and IIα Relaxation Assay *In vitro*

The human Top I and IIα inhibitory activity was determined by agarose gel electrophoresis. Reaction mixture was prepared with 0.5 μg pBR322 supercoiled DNA (TaKaRa) and human Top I (TaKaRa) or IIα (TopoGEN) enzyme in the absence or presence of compound in the Top reaction buffer (Top I: DNA Top I buffer 2 μL, DNA Top I 1U, 0.1% BSA 2 μL and sterile water up to 20 μL; Top IIα: DNA Top IIα buffer A 2 μL, DNA Top IIα buffer B 2 μL, DNA Top IIα 1U and sterile water up to 20 μL). After 30 min of incubation at 37°C, the reaction mixture was electrophoresed on 0.8% agarose gel at 80 V for 50 min with TAE running buffer. The gel was then immersed in the Genecolour I TM staining solution for 45 min and photographed under UV light.

#### Cell Cycle Assay

MCF-7 cells in logarithmic growth phase were seeded in 6-well plates (6 × 10^5^ cells/well) and incubated with different doses of compound **8a** (0, 0.15, 0.3, and 0.6 μM) for 24 h. Cells were then washed twice with cold PBS and fixed in 70% precooled ethanol at 4°C for 12 h. After the fixation, cells were washed again with PBS and stained with PI/RNase A for 30 min at room temperature, and eventually subjected to flow cytometry (CytoFLEX, Beckman Coulter). for cell cycle distribution determination.

#### Hoechst 33258 Staining

MCF-7 cells in logarithmic growth phase were seeded in 6-well plates (4 × 10^5^ cells/well) and incubated with different doses of compound **8a** (0, 0.15, 0.3, and 0.6 μM) for 24 h. Cells were then washed twice with PBS and stained with Hoechst 33258 working solution for 30 min at 37°C under 5% CO_2_. The morphological changes of apoptotic cells were observed with a fluorescence microscope (Leica DMI 4000B) with blue filter.

#### Annexin V/PI Detection

MCF-7 cells in logarithmic growth phase were seeded in 6-well plates (4 × 10^5^ cells/well) and incubated with different doses of compound **8a** (0, 0.15, 0.3, and 0.6 μM) for 24 h. After the incubation, cells were washed with PBS, collected, resuspended with binding buffer from the Annexin V-FITC kit (Thermo fisher Co., USA), and then added with 5 μl annexin V-FITC and mixed gently. After 10 min of incubation, 1 μl PI was added to each sample and mixed gently. After incubation at room temperature for another 20 min in the dark, cells were subjected to flow cytometer (CytoFLEX, Beckman Coulter).

#### Western Blotting

MCF-7 cells were incubated with different doses of compound **8a** (0, 0.15, 0.3, and 0.6 μM) for 24 h, and then total cell proteins were extracted with RIPA buffer supplemented with 1:100 protease inhibitor (info) and phosphatase inhibitor (info). Sample protein concentrations were determined with BCA assay (ComWin Biotech Co., Beijing, China), then equal amounts of protein (30 μg) were mixed with sampling buffer and denatured for 5 min at 100°C. Resulting samples were then subjected to Sodium dodecyl sulfate-polyacrylamide electrophoresis (SDS-PAGE). After electrophoresis, proteins were transferred to polyvinylidene difluoride (PVDF) membrane (Millipore) and blocked with 5% fat-free dry milk in 1 × Tris-buffered saline (TBST) for 2 h at room temperature. Membranes were then probed with Bcl-2 (rabbit, 1:1,000, Santa Cruz, CA), Bax (rabbit, 1:1,000, Santa Cruz, CA) and β-actin antibodies at 4°C overnight. The membranes were then washed with TBST three times and incubated with anti-rabbit secondary antibody (Santa Cruz, CA) and visualized with ECL-detecting reagents (ComWin Biotech Co., Beijing, China). The images were obtained from 6000 pro (Clinx Science Instruments Co., Ltd., Shanghai, China) and analyzed with Image Studio Lite software.

### Statistical Analysis

Results were expressed as mean ± standard deviation (SD) of three independent experiments performed in triplicates (*n* = 3). SPSS 19.0 software were used for statistical analysis and the means between two groups were compared by one way analysis of variance (ANOVA) with Dunnett's test, *P* < 0.05 was considered significant.

## Data Availability

The raw data supporting the conclusions of this manuscript will be made available by the authors, without undue reservation, to any qualified researcher.

## Author Contributions

WS and FY designed the project. MW, YC, QL, and QX performed the experiments. MW and LZ analyzed the data and wrote the manuscript. All authors discussed the results and contributed to the manuscript.

### Conflict of Interest Statement

The authors declare that the research was conducted in the absence of any commercial or financial relationships that could be construed as a potential conflict of interest.
